# The collection of images of an insulator taken outdoors in varying lighting conditions with additional laser spots

**DOI:** 10.1016/j.dib.2018.03.063

**Published:** 2018-03-21

**Authors:** Michał Tomaszewski, Bogdan Ruszczak, Paweł Michalski

**Affiliations:** Opole University of Technology, Institute of Computer Science, Poland

## Abstract

Electrical insulators are elements of power lines that require periodical diagnostics. Due to their location on the components of high-voltage power lines, their imaging can be cumbersome and time-consuming, especially under varying lighting conditions.

Insulator diagnostics with the use of visual methods may require localizing insulators in the scene. Studies focused on insulator localization in the scene apply a number of methods, including: texture analysis, MRF (Markov Random Field), Gabor filters or GLCM (Gray Level Co-Occurrence Matrix) [1], [2]. Some methods, e.g. those which localize insulators based on colour analysis [3], rely on object and scene illumination, which is why the images from the dataset are taken under varying lighting conditions. The dataset may also be used to compare the effectiveness of different methods of localizing insulators in images.

This article presents high-resolution images depicting a long rod electrical insulator under varying lighting conditions and against different backgrounds: crops, forest and grass. The dataset contains images with visible laser spots (generated by a device emitting light at the wavelength of 532 nm) and images without such spots, as well as complementary data concerning the illumination level and insulator position in the scene, the number of registered laser spots, and their coordinates in the image. The laser spots may be used to support object-localizing algorithms, while the images without spots may serve as a source of information for those algorithms which do not need spots to localize an insulator.

**Specifications table**

**Table:** 

Subject area	*Image processing, electrical engineering*
More specific subject area	Object localization/detection, infrastructure diagnostic and maintenance
Type of data	*Images, lighting measurements, ROI, blobs coordinates*
How data was acquired	Camera: Canon EOS 5D Mark II;
	Lenses: Canon 50 mm f/1,8, Tamron AF 28 – 300 mm
	Measuring device: Testo 435-4 + Testo 0635 0545 (lux probe for illumination levels measurement)
Data format	*JPG, CSV*
Experimental factors	*The imagery was collected in several outdoor fields areas on the ground.*
Experimental features	*Part of the dataset images were acquired with green laser spots on the insulator surface*
Data source location	*Outdoor imagery and measurements*
Data accessibility	http://cv.po.opole.pl/dataset1

**Value of the data**

The dataset will be useful:

• for the development of methods of insulator detection and localization in images.
• for the development of methods used to detect laser spots (especially in green in outdoors registered images),
• for the verification of methods used to search for laser spots in images taken outside and under varying lighting conditions.
• as a source for insulator surface assessment methods.
• as a source for deep learning algorithms.

## 1 Data

The dataset is composed of image sets grouped by scenes, and additional information:

• Insulator images with laser spots,
• Insulator images without laser spots,
• Information on illuminance in lux [lx] at the moment of taking the picture,
• Information on the localization of the insulator in the image (*x*, *y*, width, height) – for the verification of automatic localization,
• Information on the number and coordinates of laser spots in images with laser spots.

The dataset contains images of a ceramic long rod insulator, taken against several types of complex background [1]. The background is made up of scenes with the vast majority of various shades of green (such as: forest, crops, grass). The insulator was placed on a special frame, and as a result the images were obtained in conditions close to the real environment [2]. In order to verify the impact of lighting on the effectiveness of the computing methods, the images were taken in different scenes, at different times of day and in different positions (horizontal, vertical) [3]. The position of the camera and the insulator remained unchanged throughout image registration.

## 2 Experimental design, materials and methods

**Fig. 1:**
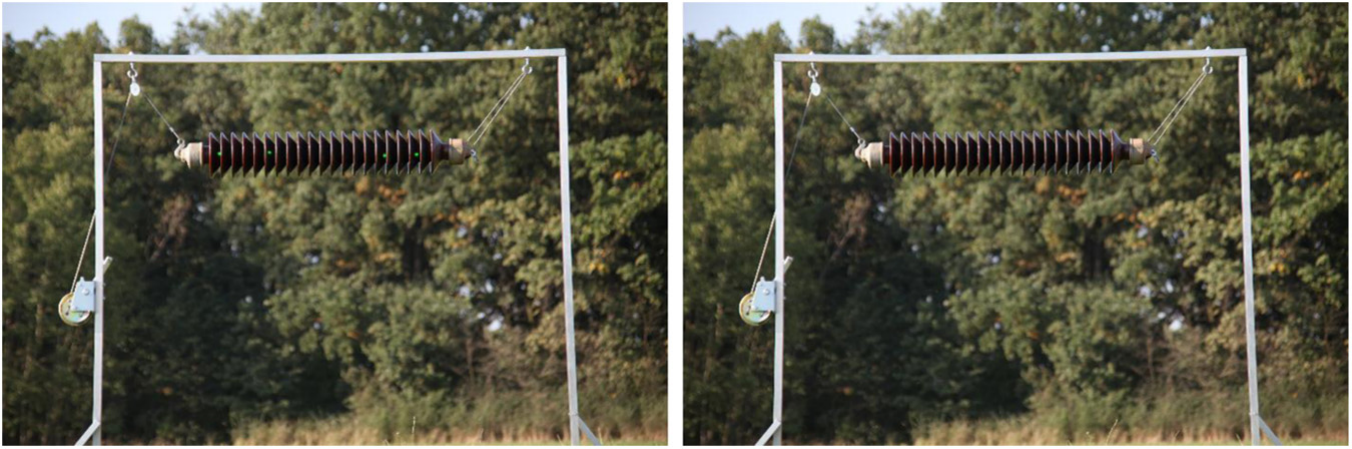
A couple of images with laser markers on the left and without them on the right.

**Fig. 2:**
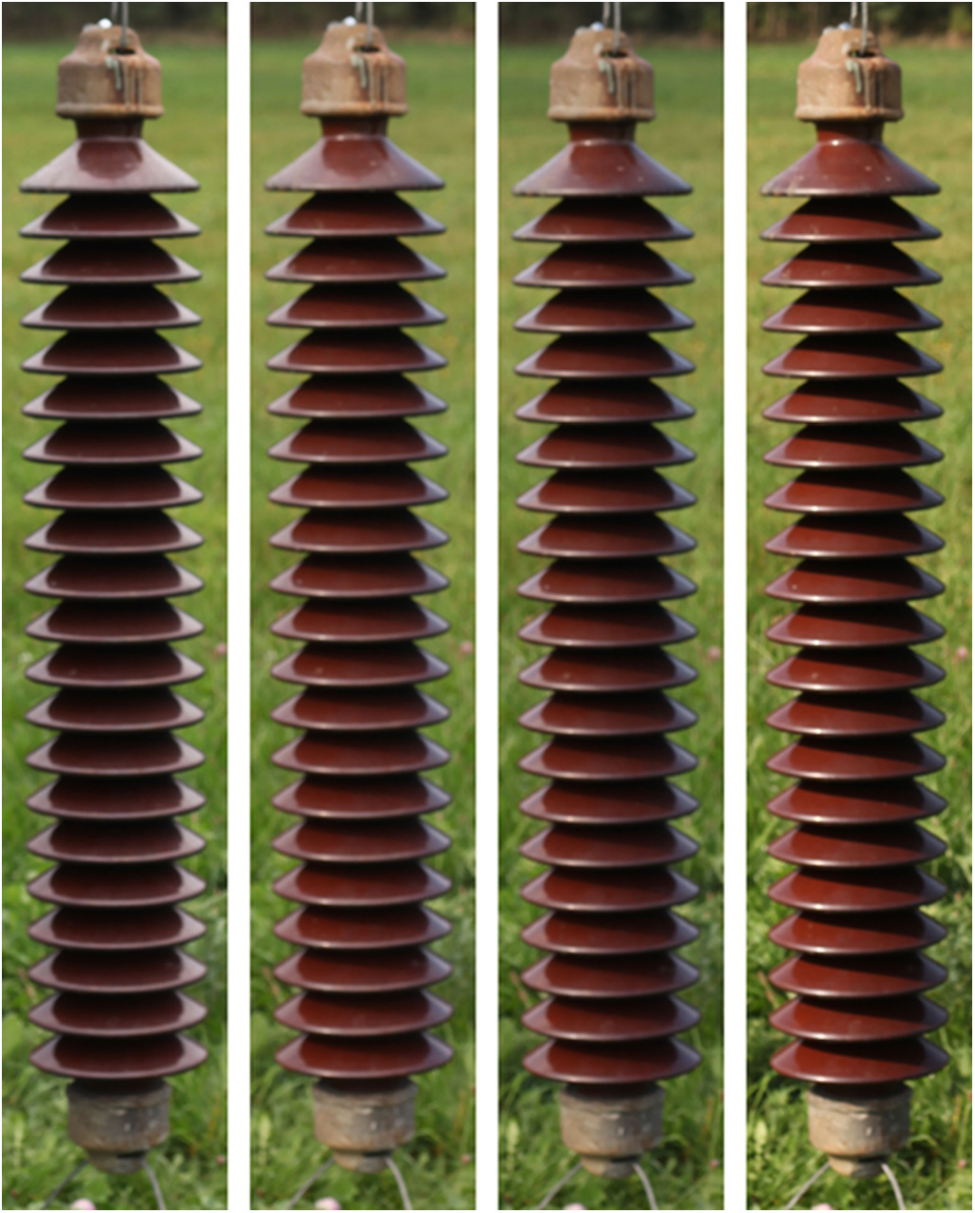
Exemplary samples of illuminance variation on the insulator surface.

**Fig. 3:**
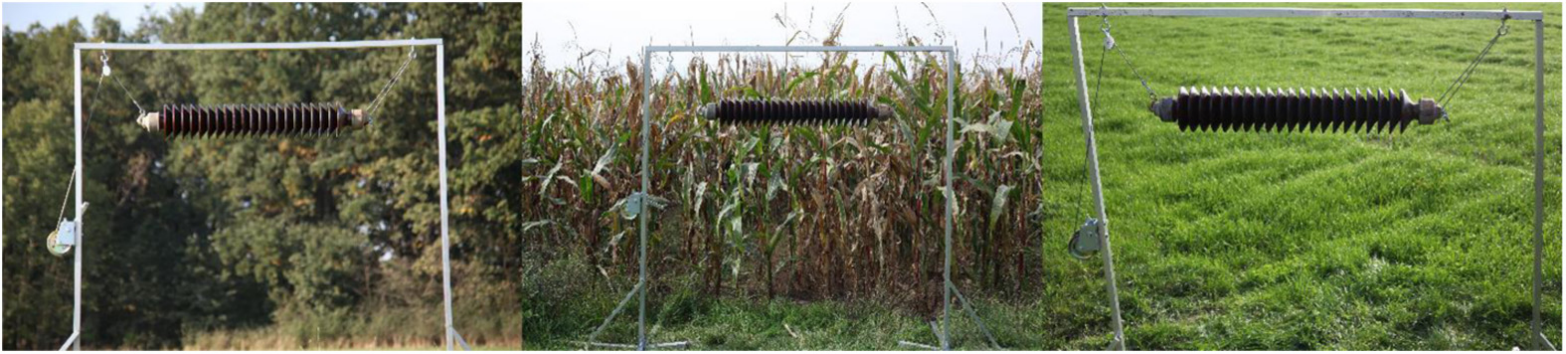
Research scenes.

The images were taken with a Canon EOS 5D Mark II camera with Canon 50 mm f/1.8 and Tamron AF 28–300 mm lenses, using an additional interval photo control system and a set of 4 laser sources with the power of 50 mW and the wavelength of 532 nm. The insulator was placed on an aluminium frame, allowing the change of its positions to obtain a desired angle. The illuminance was measured with Testo 435-4 and Testo 0635 0545 (lux probe for illumination levels measurement). The images were taken from a 5 m distance. The illuminance variation during the measurements was between 632 and 45479 lx. The images were taken with the interval alternatively with visible laser markers on the object and without them, which yielded the total of 2630 images (Fig. 1, Fig. 2, Fig. 3).
